# Understanding the Complexity of the Tumor Microenvironment in K-ras Mutant Lung Cancer: Finding an Alternative Path to Prevention and Treatment

**DOI:** 10.3389/fonc.2019.01556

**Published:** 2020-01-22

**Authors:** Shanshan Deng, Michael J. Clowers, Walter V. Velasco, Marco Ramos-Castaneda, Seyed Javad Moghaddam

**Affiliations:** ^1^Department of Pulmonary Medicine, The University of Texas MD Anderson Cancer Center, Houston, TX, United States; ^2^Department of Respiratory and Critical Care Medicine, Second Affiliated Hospital of Xi'an Jiaotong University, Xi'an, China; ^3^MD Anderson Cancer Center UTHealth Graduate School of Biomedical Sciences, Houston, TX, United States

**Keywords:** lung cancer, inflammation, cytokine, tumor microenvironment, K-ras

## Abstract

Kirsten rat sarcoma viral oncogene (K-ras) is a well-documented, frequently mutated gene in lung cancer. Since K-ras regulates numerous signaling pathways related to cell survival and proliferation, mutations in this gene are powerful drivers of tumorigenesis and confer prodigious survival advantages to developing tumors. These malignant cells dramatically alter their local tissue environment and in the process recruit a powerful ally: inflammation. Inflammation in the context of the tumor microenvironment can be described as either antitumor or protumor (i.e., aiding or restricting tumor progression, respectively). Many current treatments, like immune checkpoint blockade, seek to augment antitumor inflammation by alleviating inhibitory signaling in cytotoxic T cells; however, a burgeoning area of research is now focusing on ways to modulate and mitigate protumor inflammation. Here, we summarize the interplay of tumor-promoting inflammation and K-ras mutant lung cancer pathogenesis by exploring the cytokines, signaling pathways, and immune cells that mediate this process.

## Introduction

A staggering 23% of cancer-related deaths in the Unites States can be imputed to lung cancer, which translates to upwards of 140,000 deaths per year (1). In 90% of cases, lung cancer is caused by cigarette smoke and subsequent DNA mutations (2). One commonly mutated gene, particularly in lung adenocarcinoma (LUAD), is Kirsten rat sarcoma viral oncogene (K-ras) (3). In the wild type state, K-ras hydrolyzes GTP and activates the Raf-MEK-ERK signaling cascade leading to cell survival, cell cycle progression, and cell polarity (4, 5). However, once mutated, overactive K-ras drives these processes independently of upstream signals, thereby heavily contributing to tumorigenesis (4). Logically, efforts have been made to pharmacologically target K-ras, yet all treatments tried so far have proven fruitless (6). Although downstream effectors of K-ras signaling (e.g., MEK) are being explored as alternate targets (7), another potential therapeutic modality is emerging: targeting tumor-promoting inflammation.

**Figure 1:**
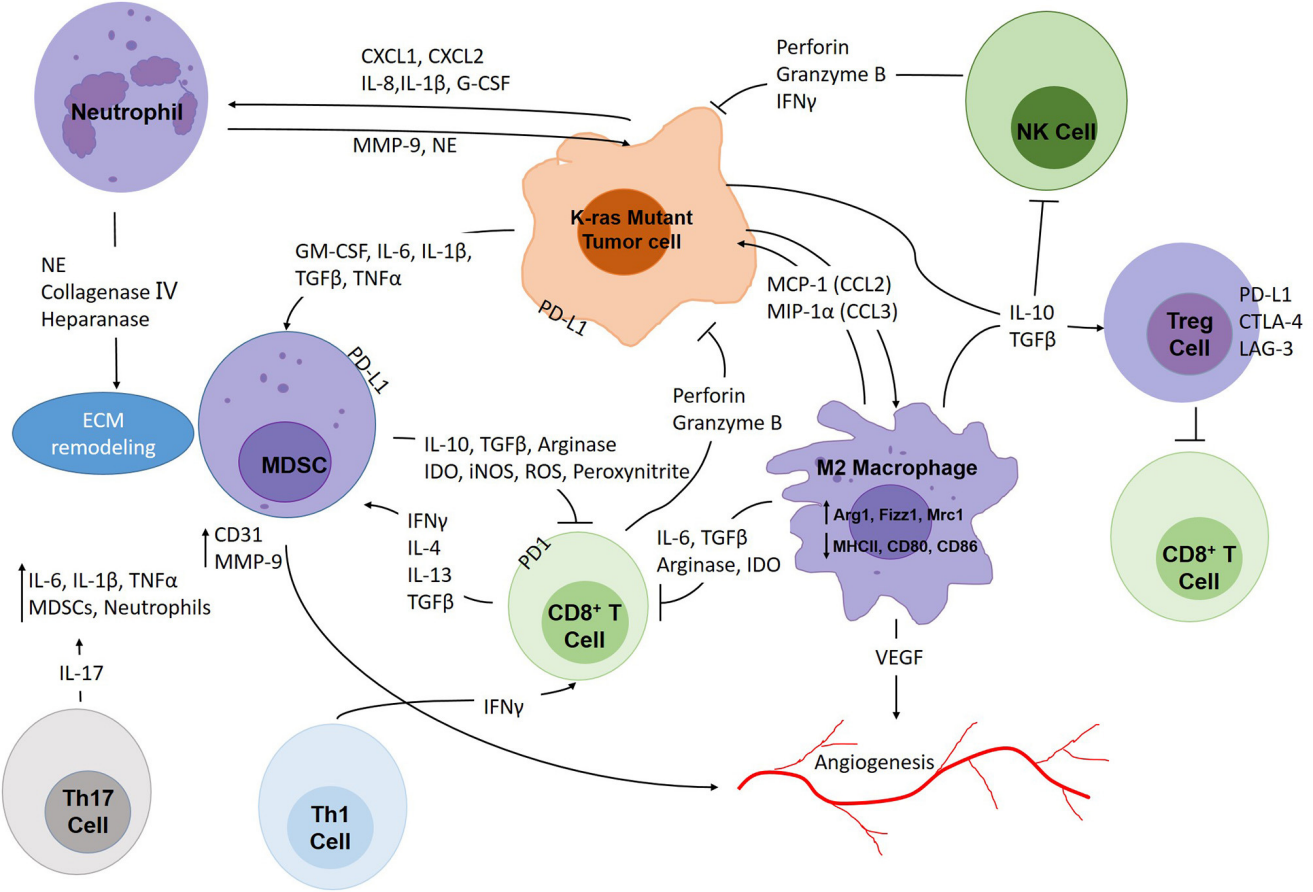
The intricate interplay within the K-ras mutant lung cancer tumor microenvironment. Tumors are constantly infiltrated with immune cells which make up important components of the TME. Tumor cells attract immunosuppressive immune cells such as M2 macrophages, neutrophils, MDSCs, Th17 and Treg cells through secretion of soluble factors (CCL2, CCL3, CXCL1, CXCL2, IL-1β, IL-6, IL-8, IL-10, TGFβ, G-CSF, etc.). These cells promote tumor growth, angiogenesis, and at the same time, protect tumor cells from cytotoxic effects. CD8^+^ T cells and NK cells attack tumor cells through secretion of perforin, granzyme B and IFNγ, and Th1 cells act as important assistants for CD8^+^ T cells. However, tumor cells, MDSCs and Treg cells could render cytotoxic immune cells incompetent through expressing immune checkpoint molecules. Moreover, immunosuppressive cells also produce soluble factors that exhaust CD8^+^ T cells and NK cells, such as arginase, IDO, iNOS, TGFβ, etc.

Unlike other oncogenic changes that have already become attractive drug targets, such as EGFR mutations and ALK, ROS and RET rearrangements, K-ras mutations are still viewed as “undruggable.” However, studies have shown that K-ras mutant lung cancer is strongly associated with inflammation which renders a new target for tackling this disease. Inflammation has been recognized as a key promoter of cancer initiation and progression (8). The immune system can often deleteriously impact antitumor responses by producing factors that aid mutagenesis, tumorigenesis, and immune suppression (8). Through the secretion of cytokines, tumor cells can reprogram the tumor microenvironment (TME), recruit immune cells, and then sway these cells toward non-productive immune responses while simultaneously dampening cytotoxic responses (9). The end result, as summarized in Figure 1, yields a protumor TME littered with molecules and cells helping the cancer survive and resist treatment.

Using genetically modified mouse models, researchers have found that lung cancers with both K-ras and EGFR mutations display immune responses with 3–5 fold increase in total infiltrating CD45^+^ cells compared with normal lung tissue (10). However, EGFR mutant lung cancers exhibit myeloid cell recruitment, but no CD8^+^ immune response was observed, suggesting that EGFR mutations may not be able to generate a sufficient antigen-driven immune response (10). In contrast, both pre- and clinical data have shown that K-ras mutant lung cancers demonstrate significant infiltration of multiple inflammatory cells, such as myeloid cells, CD8^+^ T cells, regulatory T cells (Tregs), IL-17-producing lymphocytes, and inflammatory cytokines (e.g., IL-6, IL-8, CXCL1) (10, 11). The reason why K-ras mutant lung cancer could generate a stronger CD8^+^ T cell response might be the fact that it is strongly associated with cigarette smoke and a high mutation burden that could generate an antigen-specific response. Moreover, K-ras mutations have been reported to be associated with increased PD-L1 expression (12). Patients with K-ras/Tp53 co-mutations exhibit higher PD-L1 and a higher PD-L1^+^/CD8A^+^ ratio, reaping remarkable benefits from PD-1 inhibitors (13).

In contrast with other subgroups of NSCLC which are deemed “immune-cold,” the future direction for tackling K-ras mutant lung cancer might rest on controlling tumor-promoting inflammation. In order to combat this inflammatory environment, it is necessary to take into account the main signaling pathways involved, the cytokines and other soluble factors present in the TME, and the immune cells that are recruited. In this review, we describe the interplay between these components in the TME and highlight ways in which future cancer therapeutics may target tumor-promoting inflammation in K-ras mutant lung cancer.

## Inflammatory Signaling Pathways

Signaling downstream of K-ras plays a pivotal role in K-ras-driven non-small cell lung cancer (NSCLC), especially LUAD. In fact, studies revolving around this oncogene have identified STAT3 and NF-κB as key pathways. In this section we will cover the importance of each of these pathways and their contributions to understanding the mechanisms behind this increasingly important disease.

### STAT3

An important signaling mediator downstream and associated with K-ras mutations is signal transducer and activator of transcription (STAT), particularly STAT3. STAT3 is triggered via several cytokines and growth factors; one, which we discuss later, is IL-6 (14). When IL-6 binds to its receptor, it can activate Janus kinase non-receptor tyrosine kinases (JAKs) 1 and 2 (14). These JAKs will cause receptor phosphorylation, which will produce docking sites for signaling molecules, mainly STAT3 (15). Ultimately, STAT3 will dimerize and translocate to the nucleus in order to transcribe several target genes involved in cellular processes like apoptosis, angiogenesis, and metastasis (14).

The role that STAT3 plays in lung cancer is controversial due to opposite findings by various researches. Mohrherr et al. demonstrate the presence of JAK1 and JAK2 in human LUAD that positively correlates with disease progression and K-ras activity. In their mouse models, they administered a JAK1/2 selective tyrosine kinase inhibitor called ruxolitinib. With the administration of this drug they found a reduction in tumor cell proliferation and effectively ameliorated the tumorigenic phenotype in both immunocompetent and immunodeficient mouse models of K-ras-driven LUAD (16). Another research group, Grabner et al. has also interrogated the role of STAT3 during K-ras-driven lung tumorigenesis using the Cre-inducible K-ras^G12D^ knock-in lung cancer mouse model as well as a human xenograft model. Their findings contrastingly indicate that STAT3 functions as a tumor suppressor in both models. When they genetically eliminated STAT3 in mouse lung tumors and human LUAD cell line A549, they found increased tumor growth, higher tumor grade, increased vascularization, and significantly reduced survival. These results are further supported by clinical findings where activation and expression of STAT3 was decreased in mutant K-ras patient samples compared to mutant EGFR and wild type K-ras tumors. Essentially, higher-grade tumors showed a significant reduction in STAT3 expression when compared to their low-grade tumor counterparts. In addition, low STAT3 expression had worse overall survival when compared to patients with higher STAT3 expression levels (17, 18).

Timing also contributes to the controversy of this disease. One particular study demonstrates how lung epithelial deletion of STAT3 in mice before the induction of cancer by urethane, a smoke carcinogen, resulted in increased lung tissue damage, inflammation, and tumorigenesis, while ablation of lung epithelial STAT3 after establishing lung cancer inhibited this tumorigenic process (19). These results seem to indicate that tumor heterogeneity is an important aspect in K-ras-driven LUAD. Even though K-ras mutations may share some common signaling, some K-ras mutations such as K-ras^G12C^ tumors show greater dependence on the Raf-MEK-ERK pathway compared with K-ras^G12D^ suggesting they may be more sensitive to MEK inhibitors as assessed by Li et al. (20).

While acquisition of these tumorigenic processes are often found in K-ras mutant mice, a previously unknown function of STAT3 in regards to epithelial identity and differentiation was recently discovered. This study associates aggressive tumor behavior and acquirement of mesenchymal-like phenotypes with the loss of STAT3 function, while persistent STAT3 activation bestows a differentiated epithelial morphology to cells that can affect their potential for tumorigenesis (21). Receptor-interacting serine/threonine protein kinase 4 (RIP4) is an ankyrin repeat-containing kinase that is in charge of keratinocyte differentiation and delays cell migration during wound healing. It has recently been found to be a likely regulator of tumor differentiation in LUAD and contributes to epithelial identity and differentiation (22). This study demonstrated that poorly differentiated tumors correlate with low expression of RIP4, whereas high expression correlates with better overall survival. Cells from their *in vitro* studies associate reduced RIP4 expression with elevated activation of STAT3 signaling and had an overall increased capacity for tissue invasion. In comparison, overexpression of RIP4 inhibited STAT3: after tail vein injections of RIP4-overexpressing cells, tissue invasion and tumor formation were reduced, which was restored by co-expression of STAT3 (22).

Our own group has interestingly shown a gender-specific role for lung epithelial STAT3 signaling in the pathogenesis of K-ras-driven LUAD. Decreased tumorigenesis was found in female mice lacking epithelial STAT3, yet loss of epithelial STAT3 in male littermates led to an opposite effect of enhanced malignancy, an effect driven by induction of an NF-κB-mediated IL-6/CXCL2 associated neutrophilic response and reduction of immune-mediated cytotoxicity (23). Zhou et al. used mouse models of myeloid-specific STAT3 deletion to highlight the importance of STAT3 as a major driver of myeloid-derived suppressor cell (MDSC) and macrophage pro-tumorigenic states. They found that the antitumor T helper 1 (Th1) and CD8^+^ T cells shared an inverse relationship in the development of lung cancer. Promotion of tumorigenesis was caused by induction of Tregs, inhibition of dendritic cells (DCs), and polarization of macrophages toward a pro-tumorigenic M2 phenotype due to activation of STAT3 in MDSCs and macrophages. Conversely, deletion of myeloid STAT3 boosted antitumor immunity and suppressed lung tumorigenesis (24).

A great amount of effort has gone into the development and identification of STAT3 inhibitors that can be applied in a clinical setting. The first ones developed were direct inhibitors of STAT3, which bind to the SH2 domain of STAT3, disrupting STAT3 dimerization and DNA-binding activity (25). However, their use has been limited in patients with NSCLC since studies showed issues with tolerability (26). The use of antisense oligonucleotides, most notably AZD9150, has emerged to provide an alternate approach to inhibition of STAT3 and has shown promising results when compared to direct STAT3 inhibitors as they mitigate end-organ damage and other adverse effects (27). Indeed, with the favorable safety profile and preliminary data, further evaluation of this therapy should be investigated in order to proceed to its use in a clinical setting.

### NF-κB

Another frequently activated pathway in NSCLC is the nuclear factor-κB (NF-κB) transcription factor pathway. Five members compose this dimeric transcription factor including: RelA (p65), RelB, c-Rel, p50/p105, and p52/p100 (28). These five members are capable of forming diverse homo- and heterodimers in order to variably control gene expression which is directed by signaling from cytokines, bacterial and viral byproducts, stressful stimuli, and growth factors (29). In naïve cells, the NF-κB complex is kept in a dormant state through its interaction with inhibitor of κB (IκB) proteins. IκB is phosphorylated by the IκB kinase (IKK) complex due to cytokine signaling or other relevant stimuli and afterwards undergoes rapid degradation. NF-κB subunits are freed and then released into the nucleus where they control various gene transcription targets that are crucial in cell proliferation, cell survival, inflammation, and immune responses (30, 31).

When looking at data obtained from lung cancer patients, high levels of NF-κB activation in NSCLC was significantly associated with TNM stages: In particular, NF-κB p65 expression level was significantly increased in TNM stages III and IV when compared to stages I and II (32). Additionally, the presence of nuclear RelA and cytoplasmic phosphorylated IκB (pIκB) significantly correlated with poor patient prognosis and survival (33). Song et al. have interrogated the mechanisms behind the IκB complex specifically IKKα which is essential for NF-κB activation. They found that its inhibition upregulates NOX2 and downregulates NRF2, leading to reactive oxygen species (ROS) accumulation and blockade of cell senescence which ultimately accelerates LUAD development (34). Their work demonstrates a unique pathogenesis mechanism mediated through ROS. Our own studies have likewise shown that NF-κB is activated in tumor and surrounding inflammatory cells in our K-ras-driven mouse model of LUAD (35). Bassères et al. also demonstrate that NF-κB is important in K-ras-driven tumorigenesis because the absence of p65/RelA significantly impairs K-ras-driven lung tumorigenesis. Also, inhibition of IKKβ expression stops NF-κB activation in K-ras-driven lung cells (31). The researchers further support the importance of the IκB complex by administering an IKKβ inhibitor in primary human lung epithelial cells transformed by K-ras and K-ras-mutant lung cancer cell lines. Afterwards, they tested this drug in mouse models of K-ras-driven LUAD which resulted in smaller and lower grade tumors than mice treated with placebo in conjunction with reduced angiogenesis and inflammation (31). These studies point toward targeting IKKα and IKKβ as potential therapeutic approaches for K-ras-driven LUAD.

NF-κB may originate from myeloid cells, specifically macrophages where it plays a crucial role in mediating cytokine synthesis and secretion (36). Therefore, it is reasonable to think that myeloid cell-derived NF-κB may promote lung cancer through a mechanism of inflammatory cytokine secretion, which ultimately would lead to an inflammatory microenvironment predisposed to cancer. Inhibiting NF-κB in myeloid cells significantly decreased inflammatory chemokines and cytokines that were induced by cigarette smoke including tumor necrosis factor (TNF), CCL2, CCL3, and IL-6 as well as inflammatory cell infiltrate, which is associated with reduced size and multiplicity of the lung tumors (37, 38).

NF-κB is an important signal for humans to defend themselves from environmental insults, and it has important roles in both innate and adaptive immunity. Therefore, systemic administration of NF-κB inhibitors may negatively affect the host immune response and actually be detrimental to patient health. This is why currently there is no standalone therapy and in fact combination treatments are a better alternative for lung cancer chemoprevention (30, 39).

## Cytokines and Their Receptors

Lung cancer has been associated with many cytokine signatures that aid in the survival of tumor cells. As a rule of thumb, cytokines that promote type 1 immunity are anti-tumorigenic, whereas those that lead to type 2/3 immunity or immunosuppression are typically pro-tumorigenic. The complexity of the TME response has shown us that many cytokines play different roles in tumor cell death or nurturing, and our understanding of how this complex network behaves could help us devise different therapeutic strategies. In this section, we briefly review the role of six important cytokines in the pathogenesis of lung cancer.

### Interleukin 1β

Interleukin 1β (IL-1β) is a member of the IL-1 family of proteins. Two receptors have been reported to interact with IL-1β: type I and type II IL-1 receptors (IL-1RI/II). It is believed that both receptors mediate different actions with IL-1RI as the main signal transducer and IL-1RII as a decoy factor. Upon complex formation, the IL-1RI cytosolic domain signals through different adaptor proteins such as myeloid differentiation primary response gene 88 (MYD88) and interleukin 1 receptor-activated protein kinases 1, 2, and 4 (IRAK1/2/4). These proteins help in signal transduction of several pathways including NF-κB, PI3K/Akt/PKB, MAPK, and mTOR (40, 41).

IL-1β is expressed by many cells including natural killer (NK) cells (42), macrophages (43), endothelial cells (44), neutrophils (45), T cells, and fibroblasts (46). It has been traditionally associated with the promotion of inflammation during acute and chronic tissue injuries. Many of its immunological functions include promotion of monocyte to conventional DC differentiation, macrophage polarization toward an M1-like antitumor phenotype, and activated B cell clonal expansion and differentiation into plasma cells. IL-1β secreted by activated antigen presenting cells (APCs) induces type 1 responses by increasing interferon gamma (IFNγ) producing cytotoxic T lymphocytes (CTLs) and increasing the polarization of T cells toward Th1 (47).

Despite benefiting the type 1 immune response, IL-1β often exerts adverse effects in the context of cancer. Cytokine profiling of patients with NSCLC has shown increased levels of IL-1β in tumor specimens (48). In a NSCLC cancer study of patients treated with radiotherapy, increased levels of IL-1β showed a direct correlation with worse overall survival compared to patients with lower levels (49). Mechanistically, it has been shown in different types of cancer models that IL-1β augments intratumoral immunosuppressive macrophages and increases levels of VEGF and fibroblast growth factor (FGF), supporting angiogenesis and metastasis (50, 51). Moreover, IL-1β-deficient mice show increased DC infiltration and increased CD8^+^ lymphocytes supporting antitumor cytotoxic responses (52). GATA2, a transcription factor directly known to regulate the IL-1β signaling pathway, has been shown to function as a link between IL-1β signaling and tumor cell proliferation, since GATA2 genetic depletion diminishes tumor burden and progression in K-ras-induced lung cancer models (53). Additionally, urethane-induced lung cancer mouse models, in which myeloid NF-κB was inhibited, displayed increased levels of IL-1β and cell proliferation (54).

Recently, clinical trials targeting IL-1β in patients with atherosclerosis using the monoclonal antibody canakinumab (anti-IL-1β) have shown a direct correlation with decreased incidence of lung cancer, decreased mortality, and better prognosis when compared to patients treated with placebo (55). In addition, new ongoing clinical trials for patients with NSCLC are targeting IL-1β. A phase II clinical trial (NCT03968419) is combining canakinumab with pembrolizumab (anti-PD-1) (56), and a phase III clinical trial (NCT03631199) is exploring the safety and efficacy of pembrolizumab and conventional chemotherapy with or without canakinumab.

### Interleukin 6

The glycosylated polypeptide interleukin 6 (IL-6) is the main member of the IL-6 family cytokines. Its receptor (IL-6R) dimerizes with another IL-6/IL6R complex and forms a hexameric structure that requires glycoprotein-130 (gp130, also called IL6-Rβ) membrane protein dimers (57). Trans-signaling occurs when IL-6 binds to a soluble form of IL-6R (sIL-6R) making a complex that later can bind to intramembranous gp130. Signal transduction can proceed through different pathways, with JAK/STAT (mainly JAK2/STAT3) predominating, though other pathways such as Raf-MEK-ERK, mTOR, Akt, and PI3K also play roles in cell survival, cell proliferation, and protein synthesis (58).

IL-6 expression is triggered by different inflammatory stimuli that are associated with tissue stress or damage (e.g., ROS, ultraviolet radiation, or ionizing radiation) and is mainly regulated by activation of the NF-κB pathway. IL-6 has been shown to induce immune cell recruitment, modify the TME (59), and help tumor cells with functions such as survival, apoptosis, angiogenesis, invasiveness, and metabolism. Immunological changes are also a hallmark of IL-6 expression, mainly characterized by protumoral changes with a decrease in CD8^+^ T cell responses and cytotoxicity (60), M1 to M2 macrophage polarization, induction of T helper 17 (Th17) and Treg cell responses, as well as increased migration of MDSCs. There is also evidence supporting a role for IL-6 in promoting epithelial-mesenchymal transition (EMT) (61).

Overexpression of IL-6 has been found in most cancer types, particularly NSCLC (62, 63), and has been shown to have an inverse correlation with patient prognosis (64, 65) and higher resistance to chemotherapy (66). NSCLC patients with higher TMN staging and worse prognosis exhibited higher serum levels of IL-6 compared to patients with lower disease stages (67–69). In lung cancer mouse models, blocking or deleting IL-6 reveals a delay in tumor progression and metastasis through deactivation of the IL-6/STAT3 pathway and cell proliferation regulator cyclin D1, as well as through enhancement of tumor cell apoptosis (70). Mice with K-ras mutant lung tumors treated with anti-IL-6 antibodies have shown an overall reduction in tumor burden and a shift to a TME with a less proliferative and a less protumor inflammatory context: fewer Th17 cells, fewer Tregs, and more M1-type polarization (62). Paradoxically, some models show evidence of accelerated tumorigenesis in early stages of IL-6 depletion (70, 71). However, therapeutic antibodies against IL-6R have decreased tumorigenesis and increased apoptosis in numerous human cancer cell lines synergistically with other targeted therapies such as EGFR inhibitors (71, 72). A clinical trial (NCT03337698) using tocilizumab (anti-IL-6R) is underway in patients with metastatic disease who have or have not received previous therapy with conventional chemotherapy plus anti-PD-1/PD-L1 therapy (73).

### Interleukin 8

Interleukin 8 (IL-8) is also called neutrophil chemotactic factor or CXCL8. Its receptors, CXCR1 and CXCR2, are G-protein family members expressed mainly on neutrophils but also cells like fibroblasts, neurons, and epithelial cells (74). IL-8 expression is initiated and regulated by the NF-κB pathway, with its strongest activators being IL-1β and TNF (75). It is mainly secreted by immune cells such as macrophages, neutrophils, T cells, and non-immune cells like epithelial and endothelial cells. IL-8 secretion has several physiologic functions including chemotaxis, induction of phagocytosis, degranulation of neutrophils, DC migration, and potentiation of acute inflammatory reactions (76).

Overexpression of IL-8 and its receptors have been shown to play a pivotal role in promoting tumorigenesis in several neoplasia such as gliomas (77), cervical cancer (78), colorectal cancer (79), and lung cancer (17). Its main roles comprise autocrine and paracrine alterations of the TME, with one main mechanism being the recruitment of MDSCs (80). Other roles include angiogenesis through promotion of VEGF expression (81), induction of tumor growth, and facilitation of metastasis (82). Furthermore, IL-8 plays a central role in the promotion and induction of lung cancer cell proliferation (83), which is achieved through EGFR transactivation and increased MAPK pathway signaling (84).

High levels of IL-8 have been described within tumors of lung cancer patients with K-ras mutations, correlating with decreased disease-free survival and overall survival when compared to patients with low levels of IL-8 (85). K-ras mutant IL-8-overexpressing cell lines showed a sharp decrease in IL-8 expression when treated with K-ras-shRNA and MEK inhibitors, suggesting a direct relationship between activating K-ras mutations and upregulation of IL-8 expression. In these cell lines, inhibiting IL-8 led to decreased cell proliferation and migration, further indicating a role for IL-8 in promoting K-ras mutant lung cancer (86). Similarly, studies in which the CXCR2 receptor was inhibited through the use of neutralizing antibodies or selective inhibitors have shown a decrease in tumorigenesis, suppression of neutrophil migration, and induction of apoptosis in vascular endothelial cells in K-ras mutant mice (87, 88). Additionally, several NSCLC non-K-ras mutant cell lines where shown to have an increased proliferative rate and increased growth through the action of IL-8 in an endocrine fashion (83). Furthermore, high levels of IL-8 have been associated with resistance to lung cancer therapies such as EGFR inhibitors (89), conventional chemotherapy (90), and anti-PD-1 immunotherapy (91).

### Interleukin 17A

Interleukin 17A (IL-17) is a 155 amino acid glycoprotein, which is secreted mainly by Th17 cells as a homodimer (92, 93). It can also be produced by other cells, such as CD8^+^ T cells, NK cells, type 3 innate lymphoid cells (ILC3s), γδ T cells and in some cases epithelial cells (94–96). Its receptor, IL-17R, is ubiquitously expressed and usually forms multimeric complexes (97).

IL-17-mediated responses have been shown to produce tumorigenic effects in the early stages of multiple cancers, including lung (98), gastric (99), and prostate cancer (100). Specifically, IL-17 acts to recruit MDSCs (101) through increased levels of IL-8 and encourages angiogenesis in tumor-surrounding endothelial cells and fibroblasts via production of VEGF, TGF-β, CXCL1, and CXCL5 (102). IL-17 also elevates levels of IL-1β, IL-6, and TNF, and its signaling activates the NF-κB and MAPK pathways (103, 104). Paradoxically IL-17 has also shown antitumor activity, in which IL-17-mediated responses had higher effects than Th1-driven responses in ovarian carcinomas. This antitumor response was highly dependent on high levels of IFNγ, IL-21, IL-22, and chemokines such as CCL20 (103, 105).

Several studies have shown that increased IL-17 levels in patients with lung cancer are associated with poor prognosis and higher TNM staging (106). The use of lung cancer murine models confirmed an increased infiltration of Th17 cells in tumors. These cells promoted tumorigenesis through production of IL-17 and subsequent recruitment of myeloid cells. IL-17 deletion reduced K-ras mutant lung tumorigenesis, tumor cell proliferation, and angiogenesis (98). This was associated with decreased levels of inflammatory mediators such as IL-6 and different chemokines related to neutrophil and monocyte migration such as CXCL1, CXCL2, CCL2, and GM-CSF, resulting in fewer myeloid cells (98). IL-17 has also been shown to promote metastasis of NSCLC xenograft models due to its direct association with the IL-6/STAT3 pathway (106). Recently IL-17 has been found to promote resistance to immune checkpoint blockade in lung cancer through a neutrophil-dependent modification of the lung TME (107).

### Interleukin 22

Interleukin 22 (IL-22) is classified inside the IL-10 related cytokines. It is secreted primarily by Th17 cells (108); however, other immune cells such as γδ T cells, natural killer T cells, neutrophils, macrophages, and ILC3s secrete IL-22 (109). IL-22 expression is highly dependent on the activation of aryl hydrocarbon receptor (AHR) (110) and transcription of STAT3. Signaling pathways activated by IL-22 include the JAK-STAT pathway (particularly JAK1/2) resulting in the activation of STAT1, STAT3, and STAT5 (111, 112). Other pathways activated include MAPK, mTOR, Akt, and PI3K.

Recently it has been described that IL-22 is secreted in high amounts in different cancers of the lung, liver, skin, and colon (56, 113, 114). It has been shown to be upregulated in tumor tissues and bronchoalveolar lavage fluid of patients with recurrent NSCLC, and its abrogation decreases the proliferative and migratory capabilities of tumor cells (115, 116). IL-22R overexpression in NSCLC has also been associated with poor prognosis in patients with LUAD (117). In mice, it has been shown that IL-22 also increases levels of other cytokines such as IL-6, IL-10, TGF-β, and TNF, inducing an immunosuppressive environment. Studies in a murine K-ras mutant lung cancer model have confirmed the promoting role of IL-22 in tumorigenesis and its pro-inflammatory function that is mediated through activation of different cytokines including IL-6 and IL-17 (113). It has also been shown to trigger upregulation of stemness markers (113). Increased expression of IL-22 and its receptor IL-22R1 and increased activation of STAT3 in human lung cancer cell lines mediate resistance to standard chemotherapy (118), which could be linked to the role of IL-22 in induction of stemness properties.

### Tumor Necrosis Factor

TNF is comprised of a 233 amino acid homodimeric transmembrane protein (119). It is mostly produced by immune cells such as CD4^+^ T cells, mast cells, eosinophils, neutrophils, NK cells, and macrophages (120). TNF is a cytotoxic molecule that serves as an acute phase reaction protein that chemotactically attracts neutrophils and increases levels of endothelial adhesion proteins (120, 121). Several pathways are activated by TNF including NF-κB, Akt/PKB, MAPK, and the apoptosis pathways (73, 119, 122, 123). TNF expression is ubiquitous in different organs including lung epithelial cells, fibroblasts, and stromal tissue (121).

Chronic increase of TNF has been historically related to cancer-associated cachexia due to its effects on hypothalamic structures (124), and high serum TNF levels have been associated with worse prognosis in lung cancer (125, 126). TNF increases the number of MDSCs and neutrophils in the TME, leading to increased tumor growth, angiogenesis, and amplification of inflammation (127, 128). To model these effects, murine studies have used epithelial-specific TNF overexpression and deletion using a TNF knockout mouse in a K-ras mutant model of lung cancer to show the importance of TNF in recruiting MDSCs and promoting lung cancer (129, 130). It has also been shown that TNF neutralization impairs inflammatory cell migration and angiogenesis with an overall decrease in tumor size but not number in a urethane-induced lung cancer mouse model (131). Efforts to combine TNF agonists with other cancer therapies such as 17-allylamino-17-demethoxygeldanamycin (17AAG) have been tested in several human cancer cell lines *in vitro* (132).

Currently anti-TNF therapy is being used in different types of cancers, including lung cancer, and has been shown to enhance chemotherapeutic effect by decreasing inflammation, proliferation, and metastasis (133). Clinical trials using anti-TNF with or without chemotherapy have shown prolonged disease eradication in patients with ovarian cancer (134) and breast cancer (135).

## Immune Cells

As we have previously mentioned, inflammation significantly augments tumorigenesis (8) and in the process recruits various immune cells. Tumors are constantly infiltrated with immune cells with diverse functions which make up important components of the TME. Cells with cytotoxic activities, such as CD8^+^ T lymphocytes and NK cells, generally exert antitumor functions, whereas Tregs, Th17 cells, MDSCs, neutrophils, and macrophages are usually pro-tumorigenic. Lung cancer driven by K-ras activation in the airway epithelium elicits a formidable inflammatory response distinguished by macrophagic and neutrophilic infiltration accompanied by increased chemokines like CCL2, CXCL1, and CXCL2 (11). In this section, we will discuss the function and importance of each immune cell in the pathogenesis of K-ras mutant lung cancer.

### Macrophages

Macrophages are innate immune cells resident in tissues originating from peripheral, circulating monocytes. They are involved in the detection, phagocytosis, and destruction of foreign organisms. They also present antigens to T cells and initiate inflammation through secreting cytokines that activate other immune cells. As crucial drivers of chronic inflammation, they have been reported to be involved in almost every step of cancer progression including early carcinogenesis, metastatic progression, and therapeutic resistance. Tumor-associated macrophages (TAMs) constitute the majority of infiltrating immune cells in tumors, and their presence usually negatively correlates with clinical outcome (136). In lung cancer, higher TAM density is related to worse patient survival, and patients with recurrent disease display elevated macrophage infiltrates in their primary tumors (137, 138).

Activated macrophages are classified into two phenotypes by their roles: pro-inflammatory M1 type or anti-inflammatory M2 type (139). During early tumorigenesis, M1 macrophages play a role in the elimination of more immunogenic tumor cells, whereas less antigenic tumor cells survive and skew macrophages toward the M2 phenotype to promote tumor survival and metastasis through angiogenesis, EMT, and immune evasion (136, 140–142).

In an analysis of infiltrated immune cells in subtypes of lung cancer, researchers found that LUADs with K-ras or EGFR mutations were more densely infiltrated by cells of myeloid lineage, whereas small cell lung cancers (SCLCs) were mostly infiltrated by T cells. Moreover, in K-ras mutant LUAD macrophage content increases with time (10). This finding suggests that K-ras mutated cancer cells can modulate the TME to facilitate their growth through recruiting and remodeling macrophages. Actually, lung epithelial activation of K-ras signaling led to LUAD development and pronounced pulmonary inflammation with a 14-fold increase in macrophage content and a 100-fold increase in neutrophil count (11). Further analysis showed that macrophage chemoattractants MCP-1 (CCL2) and MIP-1α (CCL3) and neutrophil chemoattractants MIP-2 (CXCL2) and KC (CXCL1) were significantly elevated, and these chemokines were secreted by K-ras activated tumor cells (11). After being recruited to tumor sites, these macrophages were reshaped into an M2 protumor phenotype (i.e., increased Arg1, Fizz1, and Mrc1 expression) to promote tumor progression (62).

Macrophages promote tumor progression through multiple mechanisms such as boosting angiogenesis, stimulating proliferation and EMT, remodeling the extracellular matrix (ECM), enhancing immunosuppression, and inhibiting antitumor cytotoxic activities (136, 143). In NSCLC patients, tumor-infiltrating macrophage density correlated significantly and positively with intratumor microvessel counts and negatively with patient survival. Recent studies using K-ras mutant LUAD models found that skewing from M2 to M1 in the TME resulted in reduced angiogenesis (143), and knocking out TLR9 in mononuclear cells led to reduced microvessel density and lower VEGF expression (144). Macrophages also undergo bidirectional crosstalk with lung cancer cells through CCR2-CCL2 and CX3CR1-CX3CL1 signaling axes to facilitate tumor growth as well as establish a nurturing tumor microvasculature and metastasis (145).

On the other hand, M2 macrophages promote an immunosuppressive TME, as remodeling from M2 to M1 results in an attenuated immunosuppressive environment and boosts the efficiency of antitumor T cell function characterized by lower PD-L1 expression and decreased levels of IL-6 and TGF-β (143). Sustained activation of NF-κB signaling led to chronic inflammation and adenoma development accompanied by increased M2 macrophage infiltration, and these macrophages induced Treg differentiation through expression of IL-10 and TGF-β (146). In a cigarette smoke-induced K-ras mutant LUAD mouse model, myeloid cells were able to promote LUAD cell proliferation, angiogenesis, IL-6/STAT3 signaling, and infiltration of neutrophils (37). Furthermore, M2 macrophages maintain an immunosuppressive TME through downregulation of MHC II and costimulatory proteins (CD80 and CD86) and increased arginase (Arg1) and indoleamine 2,3-dioxygenase (IDO) activity to prevent the activation of antitumor immunity (147).

### Neutrophils

Chemotactic factors like IL-8, which we discussed earlier, that are produced in inflamed tissues are instrumental in recruiting neutrophils. These cells are the backbone of pathogen clearance, phagocytosing their targets while releasing antimicrobial proteins, proteases, and even their own DNA into the microenvironment in an effort to mitigate infection (136, 148).

It has been widely recognized that tumor-associated neutrophils (TANs) play an important role in cancer promotion (149). High levels of neutrophil infiltration and high neutrophil/lymphocyte ratio are negatively associated with prognosis in different malignancies including lung cancer (150–152). Similar to M1 and M2 macrophages, neutrophils are also thought to exist in two distinct phenotypes, “N1” and “N2” (153); however, there are no biomarkers yet to specifically identify these two phenotypes (136). N2 neutrophils are pro-tumorigenic by influencing angiogenesis and immune surveillance, secreting various cytokines, and generating ROS (154). Under certain conditions such as TGF-β blockade, TANs can take on an antitumor N1 phenotype (149).

TANs are recruited to the TME through cytokines and chemokines secreted by cancer cells and stromal cells, such as TGF-β, TNF, and CXCL1/2/5 (155–157). TANs are a crucial component of the lung cancer TME. Actually, neutrophils comprised 20% of all CD45^+^ cells in NSCLC samples, and they were predominantly located in tumor stroma (158). Our previous studies and others using K-ras mutant LUAD models have found that neutrophils make up a significant portion of immune cells in the TME, and lung epithelial K-ras activation significantly increases neutrophil infiltration through CXCR2 ligands CXCL1/2 (11, 35, 87, 88), with neutrophil infiltration increasing with time (10).

TANs are involved in tumor initiation and progression through promoting angiogenesis, remodeling the ECM, producing ROS, and modulating immunity (149). Neutrophils can promote lung carcinogenesis through secreting MMP-9 to prevent apoptosis (159). They also secrete neutrophil elastase (NE) that activates Akt signaling to potentiate lung cancer growth (160). Neutrophils likewise promote angiogenesis through secretion of chemokines and MMPs, which can switch on angiogenesis through counteracting anti-angiogenic molecules and promoting the release of VEGF (161). NE, collagenase IV, and heparanase produced by neutrophils degrade the ECM and assist tumor cell extravasation during the metastatic process (162, 163). Neutrophils also play a major role in reshaping the leukocyte component in the TME. Using NSCLC specimens, Kargl and colleagues (158) found a strong negative correlation between neutrophils and CD8^+^/CD4^+^ lymphocytes, suggesting lymphocytes are able to suppress neutrophils. Since neutrophil-derived NE promotes cancer development, a genetic knockout of NE resulted in reduced tumor burden, delayed cancer progression, lower proliferation rate, lower angiogenesis, and decreased levels of IL-6 and TGF-β (88). On the other hand, NE is also reported to accelerate tumor growth through skewing the PI3K axis toward tumor cell proliferation (160).

IL-17 in the K-ras mutant LUAD microenvironment induces epithelial secretion of CXCL2 and G-CSF to recruit neutrophils to the TME and exert their tumor-promoting functions (98). Neutrophils also reduce T cell homing, impair anti-PD-1 efficacy, and alter angiogenesis, leading to hypoxia, and sustained Snail expression in lung cancer cells (164). Besides the intrinsic inflammation caused by K-ras mutation, extrinsic inflammation, like airway inflammation in chronic obstructive pulmonary disease (COPD), can shift inflammation from macrophage-predominant to neutrophil-predominant and significantly promote lung tumor growth, which further emphasizes the cancer-promoting role of neutrophils (35).

### Myeloid-Derived Suppressor Cells

MDCSs represent a diverse collection of immature myeloid lineage cells that together contribute to negative regulation of the immune response during cancer and chronic inflammation (165). In mice, MDSCs are recognized as CD11b^+^GR1^+^, whereas the human phenotype is CD11b^+^CD14^−^CD33^+^ (165). MDSCs can be further classified as polymorphonuclear (PMN-MDSCs) and mononuclear (M-MDSCs) (166). The expansion and activation of MDSCs are influenced by tumor cells, activated T cells, stromal cells, and active molecules released by these cell populations (165). Tumor-secreted factors such as GM-CSF, IL-1β, IL-6, TGF-β, and TNF mediate expansion of MDSCs (167–171). T cells and tumor stromal cells activate MDSCs through TLRs, IFNγ, IL-4, IL-13, and TGF-β (165).

MDSCs are present in activated states and are potent suppressors of T cell functions (165). The main mediators of MDSC immunosuppressive functions are Arg1, iNOS, TGF-β, IL-10, COX2, and IDO (166). MDSCs express high levels of Arg1 and iNOS, both of which directly inhibit T cell function (172, 173). MDSCs generate oxidative stress by producing ROS, and inhibition of ROS in MDCSs completely abrogated the inhibition on T cells (174). Peroxynitrite is a potent oxidant produced by the body, and peroxynitrite levels are elevated at sites where MDSCs and inflammatory cell correspondingly amass. Direct contact of peroxynitrite with T cells results in T cell receptor and CD8 nitration, which renders T cells unresponsive (175). MDSCs release IDO, which polarizes APCs toward a tolerant phenotype. MDSCs also express the immune checkpoint molecule PD-L1, which binds to PD-1 on T cells resulting in T cell exhaustion (176). MDSCs also promote tumor progression through interaction with other immune cells. They produce IL-10 to inhibit DC function (177), polarize macrophages toward M2 (178), and recruit Tregs via IL-10 and TGF-β (179).

MDSCs play an important part in the TME of K-ras mutant LUAD. Oncogenic K-ras induced GM-CSF is capable of activating MDSCs, and MDSCs can directly promote angiogenesis or accumulate Tregs to enhance K-ras mutant tumor growth (82, 88). COPD-like airway inflammation in K-ras mutant LUAD can increase TNF levels, which induces a robust accumulation of MDSCs. Recruitment of MDSCs results in increased angiogenic markers CD31 and MMP-9 and increased TGF-β and IL-6 levels that induce an immune-suppressive Treg response (129). It has also been shown that IL-6 inhibition in K-ras mutant LUAD significantly reduced the MDSC population, as well as levels of Arg1, IDO, CXCL1, and IL-17 in these cells, and at the same time switched the T cell phenotype from a protumor Treg/Th17 to an antitumor Th1/CD8 T cell response (62).

### T Helper 17 Cells

Th17 cells, a T helper cell subgroup that functionally differs from conventional Th1 and Th2 cells (180), have been associated with a wide range of inflammatory diseases including COPD. The role of Th17 cells varies in different cancer types and is currently uncertain due to evidences on both anti- and protumor functions. Th17 cells have been reported to play antitumor roles as their existence in patients correlates with lower clinical stages in prostate cancer (181) and longer survival in SCLC (182). In hepatocarcinoma (183) and pancreatic carcinoma (184), Th17 cells from patients could produce IFNγ and have cytotoxic activities, which also suggests an antitumor role of Th17 cells. The effects of Th17s might vary temporally, as Th17 cells have been found to increase in early stage NSCLC tumors compared with tumor-free parenchyma. However, in advanced stage NSCLC, Th17 infiltration in positive lymph nodes is inversely related to PD-1^+^CD4^+^ T cells (185). In NSCLC, Th17 cells have been widely reported to promote tumor progression: levels of IL-17, a major cytokine from Th17 cells, are significantly higher in NSCLC patients than non-cancer patients (186), and high levels of IL-17 are positively related to lymph node metastasis and advanced staging (106). On the other hand, Th17 cells might paradoxically have antitumor functions in NSCLC: high Th17 cell counts in pleural effusion are related to better survival of NSCLC patients (187), and IL-21 secreted by Th17 cells could induce the expansion of cytotoxic CD8^+^ T cells (188).

As we have previously discussed in the cytokine section, Th17 cells produce IL-17 to promote tissue inflammation and modulate the lung TME through secreting pro-inflammatory cytokines and chemokines (98). Th17 differentiation is usually triggered by IL-23 (189), and after activation, Th17 cells produce IL-17 and IL-22 to induce the production of pro-inflammatory factors including IL-1β, IL-6, and TNF (190). K-ras mutations lead to elevated IL-17-producing T cells (10). In our previous study of K-ras mutant LUAD concurrent with COPD-related airway inflammation (98), IL-17 deficient (*il17a*^−/−^) mice have lower tumor proliferation and vascular density. Reduced tumorigenesis was associated with lower infiltration of Gr-1^+^CD11b^+^ myeloid cells (MDSCs) in *il17a*^−/−^ mice suggesting Th17 cells are involved in recruiting immunosuppressive cells. Different lung cancer subtypes and oncogenic mutations might generate distinct inflammatory responses. Currently, evidence on antitumor roles of Th17 cells in K-ras mutant lung cancer still remains scarce, and more studies are needed.

### Regulatory T Cells

As the name suggests, regulatory T cells (Tregs) are T cells that play a role in regulating or suppressing the immune system and preventing autoimmune disease. Tregs are typically characterized as CD4^+^CD25^+^ and express the nuclear transcription factor FoxP3. Treg infiltration is related to the progression of lung cancers. NSCLC specimens have been found to have a significant increase in Tregs compared with non-tumor lung tissue (158). A clinical study revealed that circulating Treg numbers rose alongside disease stage and metastasis (191). Treg levels in NSCLC tumors and peripheral blood are linked to increased risk of recurrence and poor survival (192, 193).

Tregs induce immunosuppression through contact-dependent mechanisms such as expressing co-inhibitory molecules like cytotoxic T-lymphocyte-associated protein 4 (CTLA-4), PD-1, PD-L1, lymphocyte-activation protein 3 (LAG-3), CD39/73, or through contact-independent mechanisms by producing immunosuppressive molecules like IL-10, TGF-β, adenosine, prostaglandin E_2_ (PGE_2_), and IL-35 (194, 195). In mouse LUADs, Treg depletion resulted in tumor cell death and increased levels of granzyme A, granzyme B, perforin, and IFNγ in infiltrating CD8^+^ T cells, suggesting Tregs inhibit the antitumor function of CD8^+^ T cells (196). In lung tumors, Tregs are also associated with the expression of angiogenic and metastatic potentiator cyclooxygenase-2 (COX-2), suggesting Tregs are involved in tumor cell dissemination (192). It has been shown that Tregs inhibit NK cell-mediated cytotoxicity in a TGF-β dependent manner in a mouse Lewis lung carcinoma model, and depletion of Tregs restored NK cell anti-metastatic activities (197).

Tregs play an important immunosuppressive role in the K-ras mutant LUAD microenvironment. In a study of immune cells in different subtypes of lung cancer, researchers found that K-ras mutations give rise to elevated Treg populations, and they are the only non-myeloid lineage population to expand over the course of tumor development (10). In contrast to their wild-type K-ras cousins, tumor cells bearing K-ras mutations induce suppressive Tregs by enhancing the secretion of IL-10 and TGF-β. Conversely, inhibition of K-ras reduces the Treg population in K-ras driven lung tumorigenesis even before tumor formation (198). Using antibodies against CD25 and FR4 depletes Tregs and reduces K-ras induced oncogenesis (199). In a mouse model with K-ras and p53 co-mutations, an epigenetic modifier (BET bromodomain inhibitor) significantly reduced the Treg population while increasing Th1 infiltration and antitumor effect (200). Another group using a K-ras-driven LUAD model found that a DNA damage signaling kinase inhibitor, AZD6738, successfully reduced radiation-induced Treg proliferation and infiltration while enhancing CD8^+^ T cell activity (201).

### CD8^+^ T Cells and T Helper 1 Cells

CD8^+^ T cells are the most prominent antitumor cells. Activated CD8+ T cells, referred to as CTLs, exert antitumor effects by producing perforin and granzyme containing granules (202, 203). CD4^+^ Th1 cells mediate antitumor function through secretion of pro-inflammatory cytokines such as IL-2 and IFNγ, which promote T cell priming, activation, and CTL cytotoxicity (204). Optimal T cell-based antitumor immunity necessitates both cytotoxic CD8^+^ T cells and Th1 cells to improve the efficacy of the antitumor response (205). CD8^+^ T and Th1 cell infiltrations in tumors are positively correlated with better prognosis (206).

Despite being monitored by these antitumor T cells, cancer cells still manage to prosper through exploiting an immunosuppressive TME. CD8^+^ T cells have been found to have an exhausted phenotype in the TME. Exhausted CD8^+^ T cells are characterized by high levels of inhibitory receptors (PD-1/CTLA-4/TIM-3/LAG-3//BTLA/TIGIT) and production of fewer effector cytokines (IL-2/IFNγ/TNF/GzmB) that lead a diminished ability to eliminate cancer cells and their final deletion in the TME (207, 208). In the TME, immune cells such as MDSCs and tumor-associated DCs express high levels of co-inhibitory molecules that directly suppress T cell function. Tregs and M2 macrophages produce adenosine, IL-10, and TGF-β to induce T cell exhaustion (208). Th1 cell maturation involves the consecutive activation of the transcription factors STAT1, T-bet, and STAT4. Compared with peripheral blood lymphocytes, tumor-infiltrating lymphocytes (TILs) in human head and neck squamous cell carcinoma had lower Th1 differentiation and activation, which was mechanistically regulated through suppressed activation of STAT1, T-bet, and Th1 cytokine secretion by PD-1 signaling (205).

In lung cancer patients, TILs were found to have reduced levels of perforin and granzyme, suggesting the TILs were dysfunctional (209). Fewer exhausted T cells as indicated by a low PD-1 to CD8 ratio provides a favorable immune microenvironment that aids patient survival post-resection and response to immunotherapy in advanced NSCLC (210). K-ras mutant LUAD displays greater CD8^+^ T cell content than EGFR mutant LUAD; however, CD8^+^ T cell proliferation in K-ras-driven LUAD is lower, suggesting an exhausted phenotype of T cells in this context (10). K-ras activated lung cancer cells orchestrate the TME through secreting cytokines IL-6, IL-10, IL-17, TGF-β, and TNF, which can either suppress antitumor T cells directly or stimulate immune cells such as MDSCs, Th17s, M2 macrophages, and Tregs to inhibit Th1/CD8^+^ T cell function (62, 88, 98, 129), as we have described above.

### Natural Killer Cells

NK cells are the innate relatives of CTLs that likewise eliminate infected or transformed cells through cytolytic mechanisms. These cells were first noticed for their ability to kill tumor cells without any priming or prior activation, in contrast to CTLs, which need priming by APCs (211). NK cells are required for effective tumor surveillance. NK cell infiltration in tumor biopsies is a favorable prognostic marker in cancer patients (212, 213). Upon activation, NK cells attack tumor cells through releasing cytotoxic perforin and granzyme (214) and activating apoptosis in tumor cells through the production of IFNγ and TNF or via direct cell contact through death receptor-mediated pathways such as the TRAIL and FASL pathways (136, 215). NK cells also modulate the activity of other leukocytes. They produce IFNγ for Th1 priming (216) and polarize macrophages toward an M1 phenotype (217).

In normal lung tissue, NK cell counts are comparable to those of macrophages and granulocytes. However, as myeloid cells expand with LUAD development, NK cell populations remain unaltered, and in K-ras mutant LUAD, NK cell counts even decrease with time (10). NKG2D is a stimulatory receptor expressed on NK cells, which upon activation can stimulate NK cells; however, surface expression of NKG2D on NK cells decreases significantly in K-ras and p53 tumor-bearing mice but remains unchanged in EGFR mutant LUAD (164). Soluble factors that are abundant in K-ras mutant LUAD, such as MDSC-, macrophage- and tumor cell-derived TGF-β, PGE_2_, IDO, and IL-10, also inhibit NK cell function (218). Cong and colleagues found that NK cells inhibited K-ras LUAD initiation but gradually lost their antitumor effect, that this was associated with impaired metabolism caused by aberrant fructose-1,6-bisphosphatase (FBP1) expression in NK cells, and that FBP1 inhibition could partially restore NK cell function (219).

### Innate Lymphoid Cells

Innate lymphoid cells (ILCs) are newly defined members of the lymphocyte population that derive from common lymphoid progenitors but lack specific antigen receptors (220). They play important roles in tissue homeostasis and regulate the host immune response under infection and inflammation (221). ILCs can be defined into four subgroups: NK cells, which we have already reviewed, and ILC1, ILC2, and ILC3 which function like T helper cells (Th1, Th2, and Th17, respectively) (220). NK cells have been extensively studied in various cancers. Due to their relatively recent discovery, roles of others ILCs in cancer cell biology is still an emerging research field, and there has been several reviews about ILC functions in human cancers (220, 222, 223).

Reports on ILCs in lung cancer are still limited. Researchers have found that ILC1 cells remained largely unchanged in patients' NSCLC tissues compared with patients' own normal lung tissues; however, ILC2 cells decreased in NSCLC samples while ILC3 cells increased dramatically (224), suggesting that ILC2 and ILC3 cells might affect lung cancer growth. Further analysis showed that the amount of infiltrated ILC3 cells correlated with the density of intratumoral tertiary lymphoid structures, and these ILC3 cells can be directly activated by cancer cells via the NKp44 receptor. Once activated, the ILC3s secret IL-2, which stimulates expansion of tumor-specific lymphocytes; however, since ILC3s also provide an innate source of IL-22 which might favor tumor growth, their role in NSCLC still remains to be identified (224). On the other hand, ILC3s have also been found to promote tumor growth: in squamous cell lung cancer, IL-23 can convert ILC1s to ILC3s and promote IL-17-mediated tumor growth. Clinically, numbers of ILC3s and levels of IL-17 are correlated with poor prognosis. Notably, these effects did not occur in adenocarcinoma, suggesting that the roles of these cells are quite heterogeneous among cancer types (225). ILC2s have been recently reported to secret amphiregulin, an EGFR ligand, which can promote lung tumor cell growth, facilitate resistance to apoptosis (226), and even interfere with antitumor immunity through stimulating Tregs and establishing an immunosuppressive TME (227). However, a study on ILC2s found that these cells reside in the lung and produce IL-5 to recruit eosinophils that could prevent tumor metastasis to the lung (228). In all, study on ILC functions in lung cancer and K-ras mutant LUAD is still limited, and more evidence is needed to illustrate their roles in lung cancer.

### Clinical Insights

The TME is a complex system, with tumor cells and various immune cells interacting with each other to orchestrate either an antitumor or protumor immune response. Understanding and manipulating the crosstalk between different immune cells is of great importance as the subsequent immune response could strongly impact patient outcome. Clinical trials on improving therapeutic efficacy have focused on enhancing antitumor cytotoxic activity or dampening protumor immunosuppressive responses. Since there has not been any study exclusively focused on K-ras mutant LUAD, here we are reviewing studies containing NSCLC cohorts.

In regards to enhancing antitumor immunity, the most promising field of research is immune checkpoint blockade (e.g., anti-PD1/PD-L1, anti-CTLA4), which blocks inhibitory signaling and results in activation of T cell effector function; since this topic has already been widely reviewed (229–232), we will not discuss it extensively here. DCs are powerful APCs for the induction of antigen-specific T cell responses, and DC vaccines have been introduced as a new therapeutic strategy in NSCLC as they prime and activate CD8^+^ T cells (233). Takahashi et al. reported that DC vaccines pulsed with Wilms' tumor protein-1 (WT1) peptide could improve survival in patients with advanced NSCLCs (234). Ge et al. found that DC vaccines pulsed with survivin and mucin1 (MUC1) induced modest antitumor activity and improved quality of life for patients with stage I-III NSCLC (235). Lymphocyte (particularly helper T cell) abundance and activity depends on the availability of endogenous IL-2, which is severely impaired in lung cancer (236). In a Phase II randomized study, Masotti et al. found that preoperative administration of recombinant human IL-2 (rhIL-2) increased CD8^+^ T cell, T helper cell, and NK cell numbers in NSCLC patients (237).

On the other hand, researchers have been working on mitigating protumor immunity in the TME. Albeituni et al. found that yeast-derived whole β-glucan particles (WGP) could reduce PMN-MDSCs in NSCLC patient blood, which may be a potent immune modulator of MDSC suppressive function (238). In a Phase Ia study including lung cancer patients, Kurose et al. found Treg depletion by KW-0761 (a humanized anti-human CCR4 monoclonal antibody) showed possible occurrence of an immune response and suggested that KW-0761 in conjunction with cancer vaccines or checkpoint inhibitors represents an enticing effort to augment the antitumor immune response (239). IDO inhibits T cell-mediated antitumor immunity in patients, and blocking this regulatory pathway might disinhibit T cells and improve tumor clearance. In a phase I clinical study with 15 stage III-IV NSCLC patients, Iversen et al. found IDO is frequently expressed in LUAD, and targeting IDO by a peptide vaccine reduced Tregs, enhanced CD8^+^ T cell function, and prolonged overall survival by 18.2 months (240).

To date, clinical trials besides checkpoint inhibitors (CPIs) are still limited. The interaction of immune cells in the TME is a multifaceted network, and more and more studies have been focused on combination strategies such as combining different CPIs, CPI with targeted therapy, or CPI with chemotherapy. Since each tumor is highly heterogeneous in each patient, individualized genomic and immune phenotypes are crucial to customize an optimum treatment strategy and evaluate therapeutic efficacy.

## Conclusion

We wish to emphasize the intricate interplay within the K-ras mutant lung cancer TME between tumor cells and immune cells through numerous cytokines and inflammatory signaling cascades. Figure 1 provides a visual summary: in the center of this diverse network sit the tumor cells, secreting the soluble factors needed to recruit and reprogram immune cells by activation of target pathways. In this way, the tumor cells can inhibit antitumor responses and even persuade some immune cells to promote tumorigenesis. To forge future therapeutic strategies, we foresee the need for combinatorial treatments directed toward not only preventing tumor cell-intrinsic tumor initiating factors and immune suppression but protumor inflammation as well. Since the problem is multifaceted, it is only logical that the solution should be equally complex, and as personalized medicine evolves, the ability to design combined treatments tailored to the individual should become more readily attainable.

## Author Contributions

SM, MR-C, WV, and SD participated in the conception and design of the review. MR-C, WV, SD, and MC wrote sections of the manuscript. All authors contributed to manuscript revision, read, and approved the submitted version.

### Conflict of Interest

The authors declare that the research was conducted in the absence of any commercial or financial relationships that could be construed as a potential conflict of interest.
